# SEMG signal compression based on two-dimensional techniques

**DOI:** 10.1186/s12938-016-0158-1

**Published:** 2016-04-18

**Authors:** Wheidima Carneiro de Melo, Eddie Batista de Lima Filho, Waldir Sabino da Silva Júnior

**Affiliations:** State University of Amazonas, Av. Darcy Vargas, 1200, Parque 10, 69050-020 Manaus, Brazil; Federal University of Amazonas, Av. General Rodrigo Octávio, 6200, Coroado I, 69077-000 Manaus, Brazil

**Keywords:** SEMG, Multidimensional multiscale parser, Preprocessing technique, HEVC

## Abstract

**Background:**

Recently, two-dimensional techniques have been successfully employed for compressing surface electromyographic (SEMG) records as images, through the use of image and video encoders. Such schemes usually provide specific compressors, which are tuned for SEMG data, or employ preprocessing techniques, before the two-dimensional encoding procedure, in order to provide a suitable data organization, whose correlations can be better exploited by off-the-shelf encoders. Besides preprocessing input matrices, one may also depart from those approaches and employ an adaptive framework, which is able to directly tackle SEMG signals reassembled as images.

**Methods:**

This paper proposes a new two-dimensional approach for SEMG signal compression, which is based on a recurrent pattern matching algorithm called multidimensional multiscale parser (MMP). The mentioned encoder was modified, in order to efficiently work with SEMG signals and exploit their inherent redundancies. Moreover, a new preprocessing technique, named as segmentation by similarity (SbS), which has the potential to enhance the exploitation of intra- and intersegment correlations, is introduced, the percentage difference sorting (PDS) algorithm is employed, with different image compressors, and results with the high efficiency video coding (HEVC), H.264/AVC, and JPEG2000 encoders are presented.

**Results:**

Experiments were carried out with real isometric and dynamic records, acquired in laboratory. Dynamic signals compressed with H.264/AVC and HEVC, when combined with preprocessing techniques, resulted in good percent root-mean-square difference $\times$ compression factor figures, for low and high compression factors, respectively. Besides, regarding isometric signals, the modified two-dimensional MMP algorithm outperformed state-of-the-art schemes, for low compression factors, the combination between SbS and HEVC proved to be competitive, for high compression factors, and JPEG2000, combined with PDS, provided good performance allied to low computational complexity, all in terms of percent root-mean-square difference $\times$ compression factor.

**Conclusion:**

The proposed schemes are effective and, specifically, the modified MMP algorithm can be considered as an interesting alternative for isometric signals, regarding traditional SEMG encoders. Besides, the approach based on off-the-shelf image encoders has the potential of fast implementation and dissemination, given that many embedded systems may already have such encoders available, in the underlying hardware/software architecture.

## Background

The biological signal processing area has received extensive attention from the scientific and industrial communities, mainly due to demands for quality improvement, feature extraction, signal classification, and signal compression. Indeed, current medical diagnostic systems, such as computed tomography devices, are heavily based on signal processing techniques, regarding data reconstruction and analysis [1]. Besides, there are many other possible applications, in addition to diagnostic imaging: noise removal from electrocardiographic (ECG) records [2], electromyographic (EMG) feature extraction [3], using wavelet decomposition, and automated diagnosis of epilepsy through electroencephalographic (EEG) signals, based on complex classifiers [4], are some examples.

Specifically, EMG signals, which measure the electrical activity related to the contraction of muscles in human bodies [5], are of paramount importance, given that they are fundamental for clinical applications, such as muscle fatigue assessment [6], diagnosis of neurological disorders [5], and biomechanical assessment [7]. In addition, they can be used for controlling interfaces [8], which enables the use of bionic prostheses [9] and can provide computer access for disabled persons [10], or even be employed in human emotion classification schemes [11].

There are two known methods for acquiring EMG records: the intramuscular electromyography (I-EMG), which is invasive and uses needles or wires inserted into muscles, and the surface electromyography (SEMG), which employs noninvasive electrodes applied to skin surface. Although the I-EMG technique normally presents higher quality, the SEMG approach is more interesting and popular, since it does not cause any injury to the patient’s body. Regarding their probability density function, isometric SEMG signals can be considered as near Laplacian, for constant-force, constant-angle, and maximal voluntary compression (MVC) levels below 30 % (light force), and tend to Gaussian for higher force levels [12, 13].

In applications developed for comparing signal parameters and analyzing disorder development, signal databases become necessary and wireless transmission is also possible, where the EMG interface has a receiver and signals are transmitted after being captured by associated sensors [14]. In addition, EMG records are normally acquired by multiple electrodes and present long duration. This way, based on what was presented and given the need for storage and/or transmission, there have been many recent studies focused on efficient compression methods. However, besides providing compact representations, such schemes should also preserve the clinical information available in EMG records [15].

Traditional approaches to EMG signal compression generally employ one-dimensional techniques, given that the original signal itself is one-dimensional (unless multiple electrodes are handled as a multidimensional source [16]). In one of such studies, which is available in the related literature, Norris and Lovely [17] employed adaptive differential pulse code modulation, for EMG coding. In summary, the current sample is predicted by a combination of past ones and an error signal is transmitted, after being adaptively quantized. Some algorithms also attempt to extract features from EMG signals, which are then used during the encoding process. For instance, Carotti et al. [18] proposed a technique based on autoregressive models, which is able to preserve spectral features of the input signal, and worked on a scheme capable of producing acceptable distortions in signal waveforms [19], which is based on algebraic code excited linear prediction.

Transform-based schemes usually perform better than other methods [15]. Specifically, the wavelet transform has shown good results, when compared with other options, such as the discrete cosine transform (DCT) [20]. For instance, Wellig et al. [21] proposed two algorithms, based on the wavelet transform, for encoding I-EMG signals: the single-tree algorithm, which finds the best tree-structured wavelet packet bases, and the embedded zero-tree wavelet (EZW), which optimizes the encoding procedure for a target bitrate. Norris, Englehart, and Lovely [22], in turn, employed the same EZW algorithm, but for SEMG encoding. Another scheme for SEMG compression, which uses adaptive bit-allocation for wavelet transform coefficients, through an artificial neural network, was presented by Berger et al. [23]. Finally, an approach similar to the latter, but based on spectral shape models, was proposed by Trabuco, Costa, and de Oliveira Nascimento [24].

Another approach for achieving competitive results, which is based on pattern matching techniques, is known as the multidimensional multiscale parser (MMP) algorithm. In its one-dimensional version, which was proposed by Filho, da Silva and de Carvalho [25], the input EMG signal is split into segments with 64 samples, which are then approximated by elements retrieved from an adaptive dictionary.

Although the majority of available schemes for EMG compression are one-dimensional and rely on some king of transformation or parameter extraction procedure, some recent studies have proposed frameworks that depart from traditional approaches: encoding EMG signals as two-dimensional arrays, that is, images. Chaffim et al. [26] proposed the rearrangement of the input signal as a two-dimensional matrix, by splitting the original EMG record into segments that are later reassembled as image columns, which then go through a preprocessing step, in order to increase intersegment correlations. That scheme allows the use of off-the-shelf image encoders, such as JPEG2000 [27] and H.264/AVC [28], and thus rely on the compressor capability of exploiting intra- and intersegment redundancies. Melo, Filho, and Júnior et al. [29] also employed a similar scheme, but with two different preprocessing algorithms for improving intersegment dependencies: the relative complexity sorting, which reorganizes segments based on their complexities, and the percentage difference sorting (PDS), which rearranges segments based on their similarities. The mentioned schemes presented good performance, when encoding SEMG signals, by enhancing signal correlations, which consequently result in a more effective use of the available image compression tools.

Normally, image encoders applied to SEMG signal compression are based on the transform-quantization-coding paradigm, which is very efficient for smooth images [30]. However, images created from SEMG signal segments are similar to noise [26, 29], which consequently decreases the performance of the chosen encoder, due to the energy spread across a wide frequency range [31].

In the present paper, an alternative paradigm for SEMG compression is introduced, which is based on two of the already mentioned approaches: SEMG records reassembled as images, which results in the use of a two-dimensional encoding algorithm, and recurrent pattern matching through MMP, which is enhanced by adaptive dictionaries and predictive coding techniques. It is worth noticing that MMP is a good candidate for SEMG signal coding, due to its good performance with Gaussian sources [30].

Some optimizations to the MMP structure are also introduced, with the goals of providing adaptation regarding SEMG records and increasing coding efficiency: its prediction framework was replaced by that used in the high efficiency video coding (HEVC) algorithm, the initial dictionary construction and update procedures were revised, and the block number limit, in each dictionary, was changed. Besides, in order to further improve the exploitation of intra- and intersegment dependencies, a new preprocessing technique for SEMG records, rearranged as images, is presented, which is called segmentation by similarity (SbS), and the performance of the PDS algorithm [29] is also evaluated, along with three different back ends (image encoders), which were used in their commercial configurations, that is, without any modification in compression algorithms: H.264/AVC, JPEG2000, and HEVC. In summary, regarding two-dimensional commercial encoders, the main contributions are: the compression methodology, the SbS technique, and the results with HEVC.

## Methods

### Two-dimensional SEMG encoding

**Fig. 1:**
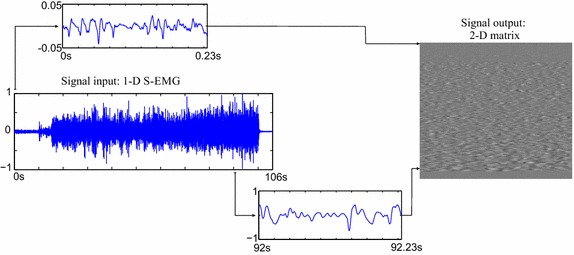
Example of a one-dimensional noninvasive electromyographic (SEMG) record rearranged into a two-dimensional array

In order to compress SEMG records using image encoders, one of the simplest approaches is to split the one-dimensional input signal into segments, of fixed or variable length, and then insert each one into a row or a column of a two-dimensional array. Later, given that the SEMG matrix was suitably assembled, an image compressor shall explore inter and intrasegment dependencies, with its available coding tools. The complete procedure is shown in Fig. 1.

Normally, image compressors used for encoding SEMG signals are based on the transform-quantization-coding paradigm [26, 29]. Indeed, that technique employs transforms for exploiting intra- and intersegment redundancies, which is followed by data quantization and, finally, entropy coding. In the quantization step, transform coefficients are quantized, which results in a smaller number of bits, and entropy coding provides an even more compact representation, by exploiting statistical dependencies regarding resulting symbols.

**Fig. 2:**
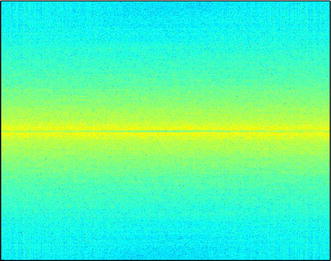
Spectrum of an SEMG image

Such methods present good performance for natural images [30], which are normally smooth and then concentrate most of their energy at low frequencies. However, images generated from SEMG signals tend to behave as noise, since their energy is more evenly distributed throughout the signal frequency range, as can be seen in Fig. 2, which tends to decrease the performance of two-dimensional encoders. In order to overcome that, two main approaches can be employed: rearranging the SEMG matrix [26], that is, adding a additional preprocessing step, with the goal of helping image encoders to better exploit signal redundancies, or choosing a two-dimensional encoder that does not expect large low-frequency content or is able to adapt to the input data [30].

Regarding the first approach, some recent studies [26, 29, 32] have proposed preprocessing techniques, which, in general, attempt to increase correlation among signal segments (columns or rows) or make them more evident, in such a way that two-dimensional coding tools can provide good performance. For instance, preprocessing techniques may rely on segment reordering [26, 29], which is based on some metric, length equalization [33], DC component removal [32], or even some kind of signal decomposition [34]. Here, two preprocessing techniques will be tackled: the newly proposed SbS, which splits the input signal adaptively, by altering the number of samples within segments, and the PDS algorithm, which rearranges segments of the input signal, based on their similarities.

The second approach may be implemented through the use of an alternative paradigm for data compression, known as pattern matching [35, 36], which is easily identified in the available image literature. The related algorithms can operate in spatial domain, where the input image is divided into blocks that are approximated by patterns available in a dictionary. In summary, those schemes can be developed without assuming any signal behavior and perform encoding in an adaptive way, which is interesting for SEMG compression.

A viable two-dimensional option is the MMP algorithm, which is based on recurrent pattern matching, uses adaptive dictionaries, and presents competitive results, for both smooth and nonsmooth images [30]. Besides, MMP has been already used for compressing ECG signals [15], stereoscopic images [37], speech signals [38], and video [39], which reinforces its universal behavior. It is worth noticing that MMP also presents good performance when used for compressing Gaussian signals [30], which, along with its universal behavior, makes MMP a good option for SEMG image encoding.

This work explores the two mentioned approaches for SEMG signal compression, in order to produce new schemes that are able to better exploit signal redundancies and provide good performance, as explained in the following sections.

### The MMP algorithm

The MMP is a data compression algorithm based on recurrent pattern matching, which was introduced by de Carvalho, da Silva e Finamore [40], in 2002, and has been updated over the years; its most recent version was named as MMP-II [30]. In the present work, the most relevant features of MMP are presented, which include its basic operation and main coding techniques.

MMP is an algorithm able to approximate image blocks at a given scale *l*, using elements from an adaptive dictionary $\mathcal D$. Actually, the latter consists of subdictionaries $\mathcal {D}^{l}$, in order to address each scale *l*. MMP starts by searching, for every block $X^l$ of the input image, an element $S_i^l$, from dictionary $\mathcal {D}^{l}$ , that minimizes the Lagrangian cost function *J*, defined by

1 $$\begin{aligned} J(X^l) = D(X^l,S_i^l)+\lambda R(S_i^l), \end{aligned}$$

where $D(X^l,S_i^l)$ is the sum of squared differences, $\lambda$ is a Lagrangian multiplier [41], and $R(S_i^l)$ is the rate necessary for encoding the dictionary element.

**Fig. 3:**
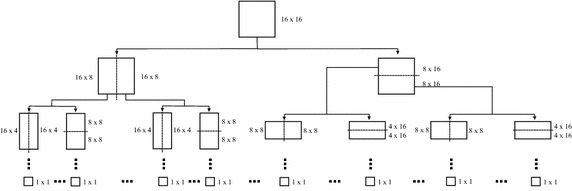
Example regarding the MMP segmentation scheme, for a block of size $16 \times 16$, which is repeated until the lowest scale is reached

After selecting the element with smallest cost, the original block is segmented across the vertical and horizontal directions [42], as shown in Fig. 3. The vertical segmentation generates two new subblocks, $$X_0^{l-1}$$ and $$X_1^{l-1}$$, each with half size, considering the original block. Then, the algorithm will look for the elements $$S_{i0}^{l-1}$$ and $$S_{i1}^{l-1}$$, in $$\mathcal {D}$$$$^{l-1}$$, that minimize the Lagrangian costs regarding $$X_0^{l-1}$$ and $$X_1^{l-1}$$, respectively. Subblocks $$X_2^{l-2}$$ and $$X_3^{l-2}$$ are generated through the horizontal segmentation and the same coding procedure is applied. Indeed, the mentioned segmentation procedure is recursively performed: MMP operates with an initial block size of $$16 \times 16$$, which corresponds to the highest scale ($$l = 25$$), and segments down until the lowest possible level ($$l = 1$$), that is, blocks of size $$1 \times 1$$. The result of such a procedure is shown in Fig. 3.

Given that block dimensions are equal to $2^m\times 2^n$, with $m,n=\{0, 1, 2, 3, 4\}$, and considering an input block of $16 \times 16$, the MMP dictionary is composed by 25 subdictionaries, at different scales. The relationship between scale and blocks, of size $M \times N$, is provided by

2 $$\begin{aligned} Scale= \left\{ \begin{array}{ll} (\log _2 M+1)(\log _2 N+1), & \text {if} \,\, M = N \\ (\log _2 M+1)(\log _2 N+1) + (\log _2 M-1)\left( \log _2 \frac{\displaystyle M}{\displaystyle N}\right) , & \text {if} \ M > N \\ (\log _2 M+1)(\log _2 N+1) + (\log _2 N-1)\left( \log _2 \frac{\displaystyle N}{\displaystyle M}\right) + 1, & \text {if} \,\, M < N \\ \end{array} \right. \end{aligned}$$

The next procedure performed by the MMP algorithm is the optimization of the segmentation tree, where it analyzes the Lagrangian cost, for each possible partition, and decides whether subblocks, at some scale, are segmented or not. For instance, if the coding cost of a parent block, at level *l*, is lower than the overall cost of its two children blocks, at level $l-1$, the parent block will not be segmented. The cost function for a block that is not segmented is defined by

3 $$\begin{aligned} J_{ns}(X^l) = J(X^l)+\lambda R(flag), \end{aligned}$$

where *R*(*flag*) is the rate needed for encoding an element *flag*, which informs that the associated block was not segmented. The Lagrangian cost regarding children blocks (subblocks) is defined by

4 $$\begin{aligned} J_{s}(X^l) = J(X_j^{l-1})+J(X_{j+1}^{l-1})+\lambda R(flag), \end{aligned}$$

where *R*(*flag*) is the rate needed for encoding the segmentation direction.

**Fig. 4:**
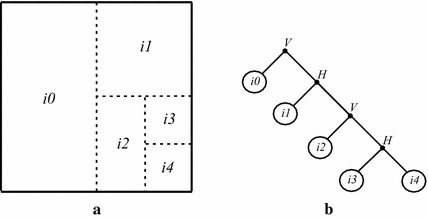
**a** Segmentation of an input image block and **b** its corresponding optimal tree

The result of the presented optimization process is the best possible representation, for the original input block, and can be represented by an optimal tree, which is obtained after pruning the full segmentation one. In the example shown in Fig. 4, every leaf of the optimal tree corresponds to a nonsegmented block, which was approximated by a single dictionary element, and every node corresponds to a segmented block, which is approximated by the concatenation of two elements available in a given dictionary.

#### Combining MMP and predictive coding

Rodrigues et al. [43] presented a new image coding algorithm called MMP-Intra, whose goal was to improve the performance of the original MMP algorithm, when compressing smooth images. Such an approach employs predictive coding techniques for exploiting spatial correlations, within input images, in the same fashion as done in H.264/MPEG-4 advanced video coding (AVC) [28]. As a result, MMP ultimately encodes residue signals with highly peaked probability distributions, which favors its block approximation procedure [30]. The same prediction modes used in the H.264/AVC standard [28] are adopted by MMP-Intra; however, the DC mode is replaced by the most frequent value (MFV) [30].

In order to generate residue blocks, MMP-Intra uses previously encoded neighboring samples for creating prediction blocks $P_M^l$, which are then subtracted from the current block $X^l$. Such a procedure is modeled by

5 $$\begin{aligned} R_{P_M}^l=X^l-P_M^l, \end{aligned}$$

where $P_M^l$ is the prediction block, at scale *l*, and *M* is the chosen prediction mode for generating the residue block $R_{P_M}^l$.

Each prediction mode has a Lagrangian cost regarding residue reconstruction, which is given by the coding cost imposed by MMP, according to (3) and (4), and the rate required to transmit the prediction mode, that is,

6 $$\begin{aligned} J_{P_M}(X^l)=J(R_{P_M}^l)+\lambda R(M). \end{aligned}$$

The MMP-Intra algorithm employs prediction techniques across the segmentation tree, in a hierarchical way. It tests each available prediction mode for every block, whose scale rages from 25 to 9, in order to find the best possible approximation. Thus, the segmentation procedure can be associated with the prediction and optimization processes.

**Fig. 5:**
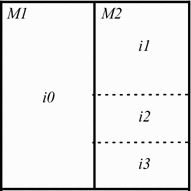
Segmentation of an image block that is encoded with prediction techniques

The final result of that procedure is represented by a segmentation tree, which is then optimized. In Fig. 5, the original block is vertically segmented and prediction procedures are performed in both halves, which generates residue blocks through prediction modes *M*1 and *M*2. The right half is also further segmented by MMP, in order to achieve an optimal representation.

#### The dictionary update procedure

The dictionary update procedure is one of the most important tasks performed by MMP, given that it is responsible for generating new matching patterns and increasing its representation power. It can be regarded as an adaptive module and is the main responsible for the universal behavior of MMP [30].

The MMP dictionary does not require any knowledge of the input signal, although that can be done, in order to improve its coding efficiency. The initial elements, of each subdictionary, are homogeneous blocks obtained within the range $[-255, 255]$, in a nonuniform fashion, except for the dictionary at scale 1 ($1 \times 1$), which includes all possible values. When a block $X^l$ is segmented, a new dictionary pattern is created, by concatenating the dictionary blocks, at scale $l - 1$, used for representing the resulting children blocks. Indeed, that new element updates every possible scale, through a separable scale transformation $T_l^s$ [40].

Every time a new element is added to the multiscale dictionary, a new index is created and, consequently, the average entropy of existing dictionary indexes increases, which reduces the MMP coding performance. Rodrigues et al. [30] introduced an efficient redundancy control scheme that limits the dictionary increase, given that a new element is added only if its relative distance, regarding existing blocks, is superior to a given threshold. Therefore, it avoids the creation of new elements that contribute little to coding.

Rodrigues et al. [30] also added geometric transforms and the additive symmetric, to the dictionary update procedure, rearranged dictionary elements within partitions with different probability contexts, according to the original element scale, and employed a norm-equalization procedure.

#### The MMP bitstream

In order to encode an optimal tree, the original MMP algorithm represents tree nodes through flags; however, due to the addition of hierarchical prediction techniques, the current tree also contains nodes for identifying segmentation procedures.  In summary, a node may be created by hierarchical prediction techniques or provided by the residue segmentation process. Accordingly, five different flags should be used for identifying nodes:

- Flag “0” represents a tree leaf, which is followed by a dictionary index; however, if a prediction mode is sent for this block, it is also followed by a prediction mode flag;
- Flag “1” represents a tree node with vertical segmentation;
- Flag “2” represents a tree node with horizontal segmentation;
- Flag “3” represents a tree with vertical segmentation, through the hierarchical prediction process;
- Flag “4” represents a tree with horizontal segmentation, through the hierarchical prediction process.

The resulting optimal tree is converted into a symbol string, through a top-down approach. When a vertical or horizontal segmentation occurs, the subtree that corresponds to the left or upper branch, respectively, is first encoded and then followed by the right or lower branch subtree. In the example of Fig. 5, MMP-II would generate the following symbol string:

$$\begin{aligned} 3 \quad \quad 0 \quad \quad M1 \quad \quad i0 \quad \quad 2 \quad \quad M2 \quad \quad 0 \quad \quad i1 \quad \quad 2 \quad \quad 0 \quad \quad i2 \quad \quad 0 \quad \quad i3. \end{aligned}$$

The generated symbols are then fed to an adaptive arithmetic encoder [30], which use distinct probabilistic models. In order to code dictionary indexes, the MMP algorithm takes into account the block scale and the element that gave rise to a respective pattern. It is done this way because the adaptive dictionary of MMP, at a given scale, is partitioned according to the scale of the block that gave rise to a given pattern.

### Optimizing MMP for SEMG signals

In this section, the contributions of the present work to the base MMP algorithm, with the goal of improving compression performance regarding SEMG signals, will be clarified.

In order to use the MMP algorithm for encoding SEMG signals and improve the associated performance, an adaptation for that kind of signal and an investigation of different predictive schemes are presented. The new prediction approach is similar to what is done in the HEVC standard [44], whose goal is to improve the exploitation of intra- and intersegment correlations.

#### Adaptation of the current MMP version (MMP-II)

MMP was developed to work with portable grayscale map images, which present 8 bits per pixel and positive dynamic range. Considering that prediction techniques may produce negative amplitudes, even when derived from grayscale levels, the dictionary is built with blocks composed by elements within $[-X, X]$. Besides, images generated from SEMG signals present a bit depth greater than 8, that is, the necessary dynamic range is different. In order to overcome those problems, MMP was adapted to handle larger dynamic ranges and thus build its dictionary according to that.

The initial dictionary elements are determined nonuniformly, as in the original MMP, but with a slight difference: specifically, the first block is the largest image value multiplied by $-1$; then, the next one is determined by the value of the previous block plus step *p*, which is defined according to

7 $$\begin{aligned} \left\{ \begin{array}{ll} p=1, & \text {if} \,\, |f(S_{0i}^{l})| \le 10 \\ p=4,& \text {if} \,\,10 < |f(S_{0i}^{l})| \le 22 \\ p=8, & \text {if} \,\, 22 < |f(S_{0i}^{l})| \le 86 \\ p=13, & \text {if} \,\, |f(S_{0i}^{l})| > 86. \\ \end{array} \right. \end{aligned}$$

As one may notice, the range of intensity values near zero is the most explored, due to the fact that the sample distribution regarding residue signals tends to be clustered around zero.

The MMP redundancy control scheme introduced a minimum distortion value *d*, among blocks of a given dictionary scale. Indeed, *d* must be carefully determined, because it will influence the resulting coding performance. For example, when it is too large, patterns can not be accurately matched, because there will be a lower number of blocks with great variation among them, which is an interesting approach for low rates. However, for high rates, lower values of *d* must be used, given that a larger number of blocks will be added to the multiscale adaptive dictionary, with small variations among them, which then allows a more accurate block matching. As the bitrate is a consequence of the numerical value chosen for $\lambda$, the function relating $\lambda$ and *d* is defined by

8 $$\begin{aligned} d(\lambda )= \left\{ \begin{array}{ll} 20, \quad \text {if} \,\, \lambda \le 4 \\ 40, & \text {if} \,\, 4 < \lambda \le 22 \\ 60, \quad \text {if} \,\, 22 < \lambda \le 50 \\ 80, \quad \text {if} \, \lambda > 50. \\ \end{array} \right. \end{aligned}$$

The relation between *d* and $\lambda$ was determined in a heuristic way, in order to optimize values for *d* and provide a good dictionary composition, for a given bitrate. In each experimental test, distinct pairs, composed of values for *d* and $\lambda$, were employed. As a result, (8) is capable of generating pairs that achieve the best results, for the entire SEMG test database, in average.

#### Improved prediction techniques

Intraframe prediction techniques have been successfully used in MMP, given that the related experimental results showed an increase in performance, for smooth and nonsmooth images. The main reason behind such an improvement is that prediction techniques change the data to be encoded, with the goal of making residue samples present highly peaked probability distributions, centered around zero [30]. Due to that, the dictionary adaptation procedure was favored, which resulted in a more efficient encoding, regarding rate-distortion figures.

Prediction algorithms basically create an estimation of image blocks, through prediction directions, which results in a difference signal that may be encoded at lower rates. Generally, prediction schemes with more prediction directions may lead to better block estimation, as long as the necessary coding rate (increased by new symbols) is compensated by the distortion gain.

**Fig. 6:**
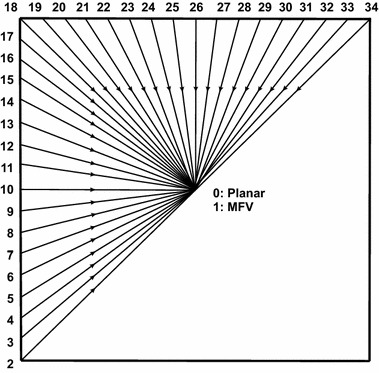
Prediction techniques (*angles*) used to enhance MMP

Based on that, the adoption of more angular predictions, as done in HEVC, is proposed in this work. In summary, the new scheme replaces the eight original directional modes, used in MMP-Intra/MMP-II, by 33 new directional modes, as shown in Fig. 6. Moreover, while the current MMP approach uses neighboring samples from previously left, above, and upper-right coded blocks, in order to perform intra prediction [30], the proposed scheme also includes lower-left coded blocks.

The MMP-II algorithm performs angular predictions based on an extrapolation regarding reconstructed samples, from previously encoded blocks, through a given direction. However, such samples may be obtained, during prediction procedures, from blocks with different scales. In order to optimize this process, two reference vectors are created, during the intra prediction procedure, which are used in all scales. The first reference, called vertical vector, contains all necessary samples to carry out angular modes 2 to 17, while the second one, called horizontal vector, contains all necessary samples to carry out angular modes 18 to 34.

In addition to angular predictions, the proposed scheme employs the planar and MFV modes, as nonangular predictions, which are commonly used for smooth regions. The MFV mode uses the most frequent value in the reference vectors, in order to compute a prediction, while the planar mode performs two linear interpolations, one using the horizontal vector and other using the vertical one; the average of those interpolations results is the predicted value.

Typically, some prediction directions may produce similar values, when used for small blocks. Due to that, it is interesting not to use prediction modes for some blocks, with small scales, in order to improve coding performance (entropy coding). Nonetheless, as SEMG images are similar to noise, small variations in prediction data may produce distorted representations. Consequently, the proposed scheme adopts the use of all possible angular prediction modes, regarding blocks at scales higher than 3; in the remaining scales, no prediction mode is used. Furthermore, the planar mode is used only in scales 4, 9, 16, and 25, because this mode has similar results regarding the MFV one, for other scales.

Since the same prediction techniques available in HEVC are used in the proposed scheme, and HEVC employs blocks of size $32 \times 32$, it may be worthwhile to investigate the use of input blocks with the same dimensions. The first consequence is the inclusion of eleven more scales (e.g., $32 \times 16$, $16 \times 32$, etc.), which would result in a coding procedure with 35 scales. Indeed, HEVC uses larger blocks, due to the need for handling high definition, which normally presents larger smooth areas. However, this is not the case with images generated from SEMG signals, since, as already mentioned, they tend to present a behavior that is still similar to noise, no matter the chosen segment size. As a consequence, matching larger blocks is still difficult, which results in further block partitioning (more segmentation and prediction mode flags) that consequently decreases the expected coding efficiency.

Another important issue regarding the use of larger blocks is the resulting coding complexity. More scales result in more dictionaries and consequently greater number of operations, since the selection of a dictionary element involves a search for the block that causes the lowest distortion. Furthermore, for each block segmentation, prediction is performed for the new children blocks, which involves operations with 33 angular and 2 nonangular prediction modes.

Based on what was presented, one can easily conclude that larger blocks would result in higher computational complexity and, for images generated from SEMG signals, the resulting coding efficiency is expected to decrease. Indeed, such conclusions were confirmed by experimental results, which were performed during the development of this work. Consequently, the largest scale for encoding SEMG signals remained as 25, that is, input blocks of $16 \times 16$.

The final result of the modifications performed in MMP-II is a new encoder, which is able to quickly adapt to nonsmooth-signal characteristics and has the potential to efficiently exploit the similarities present in SEMG images.

### Preprocessing techniques

SEMG signals may present nonstationary behavior [45], which means that the initial segmentation procedure, employed for creating image columns, may produce a two-dimensional signal with low correlation among samples from different segments, or even regarding the same segment.

However, preprocessing techniques can reshape or reorganize signals, in order to increase intra- and intersegment correlations. For instance, reordering signal segments may lead to more homogeneous areas within a SEMG image, which contributes to an increase in intersegment dependencies. In addition, preprocessing techniques can also segment input signals, according to the correlation among neighboring samples, which would directly increase intrasegment dependencies. Therefore, preprocessing techniques have the potential to improve the exploitation of signal redundancies, when taking into account image encoders.

#### The percentage difference sorting algorithm

Since images are generated from one-dimensional SEMG signals, adjacent signal segments may present low correlation, which tends to decrease the performance of image encoders. Nonetheless, each SEMG segment can be treated as an independent unit, which means that segments can be rearranged, in order to increase correlations associated with two-dimensional signals.

Given that, this paper employs a technique that reorders segments based on their similarities, known as the PDS algorithm [29], which computes similarities through

9 $$\begin{aligned} PD(x,m) = \frac{\sum\nolimits _{n=0}^{N-1}(x[n]-m[n])^2}{\sum\nolimits_{n=0}^{N-1}x^2[n]}, \end{aligned}$$

where *PD*(*x*, *m*) represents the percentage difference regarding *x* and *m* segments, *x*[*n*] is the last sorted segment, *m*[*n*] is the segment under analysis, and *N* is the number of samples in each segment.

**Fig. 7:**
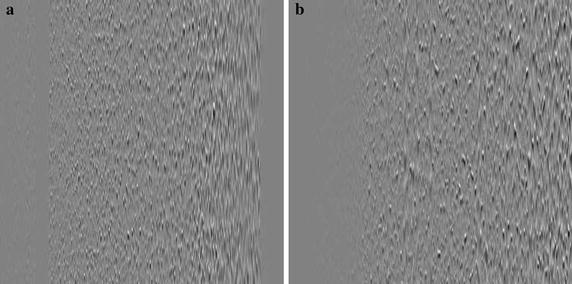
Result of percentage difference sorting: **a** original image and **b** reordered image

The PDS procedure begins by finding the segment with smallest variance, which is inserted into the first column of the SEMG matrix; the next columns are then rearranged, according to their percentage difference relative to the last sorted element. An SEMG image rearranged through PDS is shown in Fig. 7. It is possible to notice that the new representation presents a more organized texture, whose complexity increases from left to right.

It is worth noticing that when PDS is used, it is necessary to transmit the original segment positions within an SEMG image, in order to rebuild the original one-dimensional signal, during the decoding process.

#### The adaptive segmentation procedure

As mentioned earlier, in order to compress SEMG signals with image coders, one needs to split the input signal, so that the resulting segments are inserted into matrix columns. However, the way segments are chosen and also the number of samples, in each segment, usually influence the resulting intra- and intersegment dependencies, which may compromise the exploitation of signal correlations.

Another important aspect is the need for transmitting side information. Although reordering techniques have the potential to improve the efficiency of two-dimensional compressors, the requirement of transmitting original segment positions may decrease the obtained coding gains, which arose by better exploiting signal correlations. In other words, the influence of the added side information lies on the need for making the sum between the new compressed-file size (whose original SEMG matrix was rearranged) and the side-information amount lower than the original compressed-file size, for the same quality. Thus, if the amount of side information is high, the achieved coding gains, due to the increase in signal correlations, may not compensate the transmission loss regarding side information, which makes a given preprocessing procedure too expensive.

In this context, if the segmentation procedure is adaptively performed, the need for segment reordering may be reduced. Therefore, the next proposed preprocessing technique seeks the optimal number of samples within a signal segment, in order to improve both intra- and intersegment correlations, which is called segmentation by similarity.

Here, (9) (the same used for the PDS technique) is used to compute similarities between adjacent segments. The proposed procedure begins with a segment of *N* samples, which is used for computing the percentage difference between adjacent segments. Then, the average value, regarding percentage differences among segments, is obtained and stored.

The explained procedure is recursively performed, where the number of samples within a segment is given by $N=16n$, with $n=\{2,3, \ldots ,64\}$. Then, the segment size *N*, employed for creating SEMG images, that provides the lowest average percentage difference value is chosen.

One may notice that such a procedure may encounter difficulties when applied to real-time SEMG compression, due to the need for computing many possible configurations. However, if the target SEMG records already exist, an offline encoding is feasible and may lead to good results, given that the proposed technique improves the generation process of two-dimensional SEMG matrices, which produces a representation that enhances the exploitation of signal dependencies, without the need for sending additional side information.

### The compression architecture

**Fig. 8:**
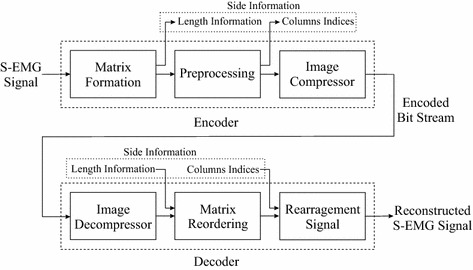
The proposed compression scheme

In the proposed methodology, the SEMG encoding is performed in three stages: matrix formation, preprocessing, and image compressor, as shown in Fig. 8. The matrix formation stage splits an input one-dimensional SEMG signal, in such a way that a matrix with *K* rows and *L* columns is built and each segment occupies one of its columns. The number of samples in the input SEMG record is then sent as side information, in order to allow the decoder to correctly reconstruct the original signal. Next, the resulting matrix is preprocessed (e.g., reordering, length equalization, etc.), with the goal of increasing signal correlations, which will probably result in a good performance regarding the two-dimensional coding step. Finally, the resulting matrix signal is encoded by an image compressor. At the decoder end, the mentioned steps are just performed in reverse order, according to what was done during encoding (see Fig. 8).

It is worth noticing that if the PDS algorithm is used, which happens in the preprocessing step, a segment size is firstly defined in the matrix formation step, in order to generate a square matrix, and a list with original column indexes is created, arithmetically coded, and then added to the file header. Besides, if the SbS technique is employed, the first two stages are performed in only one step, given that the matrix is already created with a column length (and consequently number of rows) adapted to the current signal features. As a result, no further preprocessing is required. Finally, if MMP is employed, no preprocessing is performed.

Although it was developed for SEMG signals, the proposed compression scheme is able to encode other biological signals, by employing different preprocessing techniques and image compressors. Besides, different biological signals may benefit from specific tools for their compression. For instance, ECG signals may be segmented, based on their QRS complexes, and then inserted into matrix rows, through a length equalization procedure [46].

### Database

**Fig. 9:**
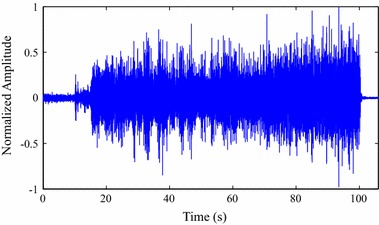
SEMG signal retrieved from the test database

The evaluation of the proposed method was carried out by experiments with real isometric and dynamic SEMG signals, acquired in laboratory. A sample SEMG signal, retrieved from the ones used in the experiments, is shown in Fig. 9.

The isometric records were collected from the biceps brachii muscle of 13 volunteers, during isometric contractions. The volunteers were sat, with their forearm parallel to their torso and sustaining an MVC of 60 %. The resulting signals were sampled at 2000 Hz, quantized with 12 bits, and present durations ranging from 1.3 to 3.0 minutes.

The dynamic records, in turn, were collected from the vastus lateralis of 13 volunteers, when performing knee-joint controlled exercises in the isokinetic concentric mode [47], through an isokinetic dynamometer, as described by Schwartz et al. [48, 49]. The resulting signals were sampled at 2048 Hz, quantized with 12 bits, bandpass filtered in the range 10–500 Hz, and present durations about 4 min.

### Performance metrics

The distortion level of reconstructed signals, after decoding, is measured through the percent root-mean-square difference (PRD) and compression factor (CF) metrics, which are commonly adopted in the related literature. The equations that define the PRD and CF figures are, respectively,

10 $$\begin{aligned} PRD = \sqrt{\frac{\sum\nolimits _{i=0}^{N-1}(x[i]-\hat{x}[i])^2}{\sum\nolimits _{i=0}^{N-1}x^2[i]}} \times 100, \end{aligned}$$

where *x*[*i*] is the original signal, $\hat{x}[i]$ is the reconstructed one, and *N* is the number of samples, and

11 $$\begin{aligned} CF = \frac{B_o-B_c}{B_o} \times 100, \end{aligned}$$

where the $B_o$ is the total number of bits, in the original signal, and $B_c$ is the total number of bits, in the compressed format. For every SEMG signal used in the performed experiments, $B_o = 12 n$, which represents 12 bits resolution and *n* samples, for each signal.

## Results and discussion

In this work, the JPEG2000, H.264/AVC (intra mode), HEVC (intra mode), and the proposed modified two-dimensional MMP encoders were used in the image compression block. The JPEG2000 encoder was retrieved from Kakadu software [50] and employs a quantization step of 0.000025 and 16 bits per sample. The JM 18.2 reference software [51] of the H.264/AVC standard was used to encode signals in FRExt High Profile 100, with deblocking filter, rate-distortion optimization, and context-adaptive binary arithmetic coding enabled. The HM 9.1 reference software [52] of the HEVC encoder is employed in high efficiency mode, with rate-distortion optimized quantization and deblocking filter enabled. Finally, both SbS and PDS are employed in the preprocessing step, unless MMP is used.

### Results for the isometric protocol

**Fig. 10:**
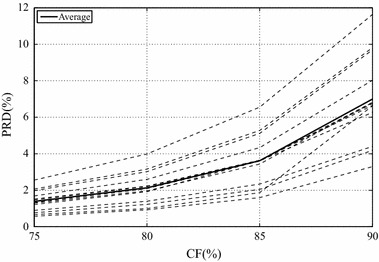
Performance of the modified two-dimensional MMP encoder with isometric signals

**Fig. 11:**
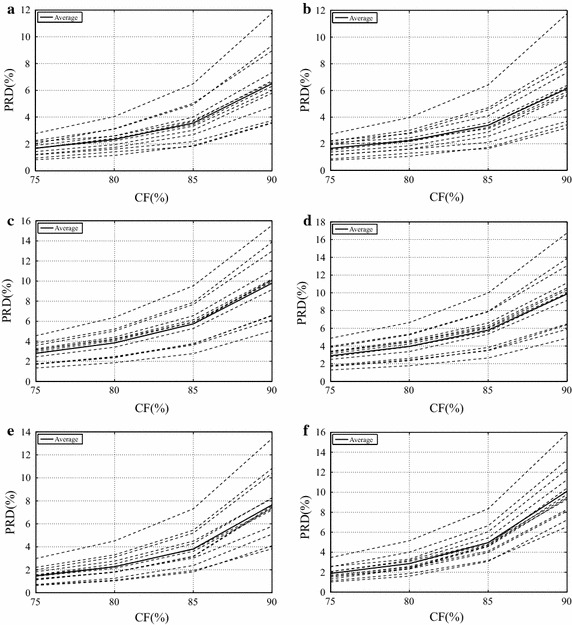
Performance of the proposed scheme regarding isometric signals, for **a** PDS and HEVC, **b** SbS and HEVC,** c** PDS and H.264/AVC,** d** SbS and H.264/AVC,** e** PDS and JPEG2000, and** f** SbS and JPEG2000

Results regarding the mentioned database (13 isometric SEMG signals), which was compressed with the proposed schemes, by employing the modified two-dimensional MMP alone and also combinations between the chosen commercial image encoders (JPEG2000, H.264/AVC, and HEVC) and the presented preprocessing techniques, are shown in Figs. 10 and 11, along with average values in Table 1. As from now, each combination will be represented by the following rule: *encoder*+*preprocessing technique*.

**Table 1:** Average PRD (%) results for the proposed methodology, regarding isometric signals

	Compression factor			
	75 %	80 %	85 %	90 %
JPEG2000	1.53	2.38	4.05	8.27
JPEG2000 + PDS	1.46	2.26	3.81	7.65
JPEG2000 + SbS	1.88	2.86	4.95	10.09
H.264/AVC	2.93	4.03	5.99	10.21
H.264/AVC + PDS	2.79	3.83	5.73	9.79
H.264/AVC + SbS	2.89	3.95	5.78	9.87
HEVC	1.71	2.33	3.56	6.47
HEVC + PDS	1.67	2.32	3.55	6.49
HEVC + SbS	1.65	2.23	3.38	6.14
Mod. 2-D MMP	1.38	2.13	3.62	7.00

The modified two-dimensional MMP achieved PRDs below 7 %, for CFs smaller than 85.4 % (see Fig. 10), which generally does not affect the diagnosis information contained in biological signals [25]. MMP also overcame HEVC, in average, even with the proposed preprocessing techniques added to the latter, for CFs lower than or equal to 80 % (see Table 1), and all other schemes, for the entire CF range (see Table 1). Although MMP and HEVC present similar prediction and input signal segmentation procedures, the fundamental difference is in the residue signal coding step, given that MMP is more successful in approximating noncoarse image blocks, that is, when low CFs are chosen.

Regarding JPEG2000, H.264/AVC, and HEVC, HEVC + SbS provided the best results, with PRD values below 7 %, for CFs below 85.5 % (see Fig. 11). The use of such an encoder and PDS produced PRD values below 85.45 %, for CFs below 85.45 %, however, one may notice that the SbS technique resulted in curves that are clustered at lower rates. Indeed, the SbS technique is more suitable to the prediction tools available in HEVC, given that a high overall correlation is obtained and the increased amount of available prediction directions is able to better exploit that feature. Besides, the difference in performance between PDS and SbS increases with higher CFs (see Fig. 11; Table 1).

JPEG2000 is competitive, however, when it is combined with SbS, there is a decrease in performance, given that the obtained results are worse than the ones provided by JPEG2000 alone (see Fig. 11; Table 1). Indeed, the SbS technique creates SEMG images that are difficult to encode, when processed by JPEG2000, mainly due to the shape of resulting homogeneous regions, which does not favor its algorithm (e.g., wavelet decomposition, etc.). Besides, although an advanced coefficient-coding scheme is used in JPEG2000, wavelet transforms generally present high performance for energy content clustered at low frequencies, which is not possible with SEMG images, as already explained.

JPEG2000 achieved PRD values lower than 7 %, for CFs below 84.6 %, when combined with PDS, and the same performance for CFs below 82.9 %, when combined with the SbS technique (see Fig. 11). The superior performance provided by PDS is again closely related to the resulting signal shape: there are large and approximately square homogeneous regions.

H.264/AVC presented the worst results for almost the entire CF range, with or without preprocessing techniques. Indeed, it may seem strange, given that there are some similarities with HEVC and what was integrated to the modified version of MMP. Although H.264/AVC, HEVC, and MMP present, in their schemes, input signal segmentation and prediction steps, H.264/AVC employs more restricted segmentation and prediction schemes, which was of paramount importance to the obtained performance.

H.264/AVC + SbS resulted in PRD values lower than 7 %, for CFs below 80.6 %, whereas the PDS technique provided such PRD values with CFs below 81 % (see Fig. 11). The slightly better performance provided by PDS is due to the restricted partitioning scheme provided H.264/AVC, which benefited from the resulting homogeneous regions.

The results obtained with PDS and JPEG2000 reveal an enhanced performance, when compared with the original encoder alone, which is also true for H.264/AVC (see Table 1). Regarding HEVC, the PDS technique provides enhanced performance for CFs lower than 90 % (see Table 1), when compared with HEVC alone. Indeed, for high compression factors (≥90 %), the increase in signal correlations did not compensate the disadvantage of sending side information.

Based on what was presented and the associated results, one may argue that the SbS technique has the potential to favor compressors that perform a flexible segmentation/prediction of the input image, as done in HEVC, while PDS is more suitable to compressors that rely on large homogeneous regions. Nonetheless, it is clear that HEVC + SbS is the best choice for high CFs and the modified MMP is more suitable to low CFs; however, the JPEG2000 + PDS is still competitive. Indeed, given that JPEG2000 presents low computational complexity, when compared with HEVC, it may be an interesting approach for low power or restricted devices.

Finally, one can argue that the modified two-dimensional MMP is very competitive and is even able to outperform transform-quantization-coding based approaches, when low CFs are chosen.

Table 2 provides results regarding MMP, H.264/AVC, JPEG2000, HEVC + SbS, JPEG2000 + PDS, H.264/AVC + PDS, and other schemes present in the available literature. Some of them employ wavelet transform and a processing step for coding transform coefficients.

**Table 2:** Average PRD (%) results for isometric SEMG signal compression, with different schemes

	Compression factor			
	75 %	80 %	85 %	90 %
Norris et al. [22]	3.8	5	7.8	13
Berger et al. [23]	2.5	3.3	6.5	13
Filho et al. [25]	1.61	2.51	4.13	7.36
Chaffim et al. (JPEG2000) [26]	3.50	4.48	6.92	13.44
Chaffim et al. (H.264/AVC) [26]	5.37	6.90	9.93	16.62
Trabuco et al. [24]	2.22	2.52	3.31	6.88
JPEG2000	1.53	2.38	4.05	8.27
JPEG2000 + PDS	1.46	2.26	3.81	7.65
H.264/AVC	2.93	4.03	5.99	10.21
H.264/AVC + PDS	2.79	3.83	5.73	9.79
HEVC + SbS	1.65	2.23	3.38	6.14
Mod. 2-D MMP	1.38	2.13	3.62	7.00

The one proposed by Norris, Englehart, and Lovely [22] encodes wavelet transform coefficients through the EZW algorithm, which exploits correlations between subbands and encodes coefficients according to their order of importance. The scheme proposed by Berger et al. [23] quantizes transform coefficients through a Kohonen layer, in order to determine the coefficient bit allocation. Likewise, Trabuco, Costa, and de Oliveira Nascimento [24] use a dynamic bit allocation scheme based on mathematical models for spectral shapes, where one is the rotated hyperbolic tangent. A scheme similar to what is proposed here was developed by Chaffim et al. [26] and employs a preprocessing technique called correlation sorting, which is combined with the H.264/AVC and JPEG2000 encoders. Actually, their main differences lie on the developed preprocessing techniques and encoder configurations. Finally, Filho et al. [25] proposes a one-dimensional approach of MMP, which uses an adaptive dictionary that is updated by concatenations, expansions and contractions of previously encoded blocks.

The test signals used in the other evaluated methods present similar acquisition protocols, regarding what was employed in the present work. Besides, almost all signals were acquired during isometric contractions of the biceps brachii muscle, with 2 kHz samples per second and 12-bit resolution, except for the one presented by Trabuco et al. which uses 16-bit resolution. As a result, such facts indicate that the performance comparison performed here is valid and, regarding Trabuco et al., at least a rough analysis may be carried out.

The proposed modified two-dimensional MMP achieved the best performance for CFs of 75 and 80 %, overcoming transform-quantization-coding schemes with wavelets and DCT, and also a one-dimensional version of MMP. Indeed, the latter was already expected, since the proposed modified two-dimensional MMP encoder has a lot of additional tools, such as prediction techniques, norm-equalization procedure, and dictionary-redundancy control, and is more successful in exploiting intra- and intersegment correlations. In that same low-CF range, JPEG2000 + PDS overcame the one-dimensional MMP and HEVC + SbS presented competitive results.

Regarding CFs of 85 and 90 %, the proposed schemes provided competitive results, however, even HEVC + SbS was outperformed, at a CF of 85 %, by the method proposed by Trabuco, Costa, and de Oliveira Nascimento [24]. The main reason is that the redundancy exploitation regarding transform coefficients, by the dynamic bit allocation employed in the latter, was more effective. Particularly, the mathematical quantization model of that method approximates the spectral shape of the signal, which, for that CR, was more efficient than the adaptive dictionary of the proposed MMP version.

Finally, the performance of JPEG2000 and H.264/AVC, with and without the proposed preprocessing techniques, overcame the method proposed by Chaffim et al. [26], which uses the same encoders, but with different preprocessing techniques. Indeed, it seems that even the conversion from one-dimensional SEMG records to SEMG images and the basic compression framework, presented here, were more carefully performed and are somehow superior, or the chosen image encoders were not properly configured. Moreover, it is important to note that the results achieved by HEVC + SbS were superior to all other evaluated methods, regarding CFs of 80 and 90 %, but the proposed modified two-dimensional MMP, at a CF of 80 %.

**Fig. 12:**
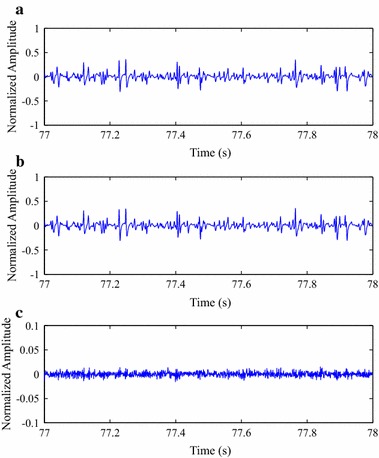
Compression result for a segment from one of the test isometric SEMG signals, at a CF of 87.3 %:** a** original,** b** reconstructed after being encoded with the modified two-dimensional MMP, and** c** error signal

In order to illustrate the quality of reconstructed isometric SEMG signals, after compression processes with MMP, original and reconstructed signal segments are shown in Fig. 12, as well as the associated error signal. As one can notice, the visual differences between the original and reconstructed signals are not meaningful, that is, the error-signal energy is low. Thus, in that case, it can be stated that the spatial domain approach performed by the proposed MMP algorithm has the potential to preserve the input signal waveform and, consequently, its clinical information.

**Fig. 13:**
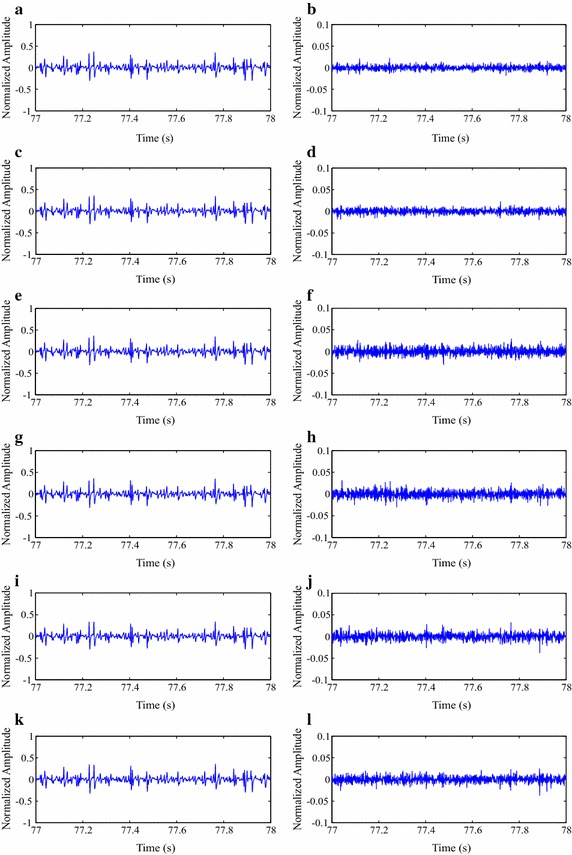
Compression result for a segment from one of the test isometric SEMG signals, at a CF of 87.3 %:** a** signal reconstruction with SbS + HEVC, and** b** error signal,** c** signal reconstruction with PDS + HEVC and** d** error signal,** e** signal reconstruction with SbS + H.264/AVC and** f** error signal,** g** signal reconstruction with PDS + H.264/AVC, and** h** error signal,** i** signal reconstruction with SbS + JPEG2000, and** j** error signal, and** k** signal reconstruction with PDS + JPEG2000, and** l** error signal

The same conclusions presented in the last paragraph can be used for Fig. 13, whose reconstructed and error signals are related to the proposed image encoders and preprocessing techniques, with the same input signal and CF. Moreover, as the performance of each association between preprocessing technique and encoder is different, the resulting error signals are also distinct, although they present a scale that is ten times smaller.

### Results for the dynamic protocol

**Fig. 14:**
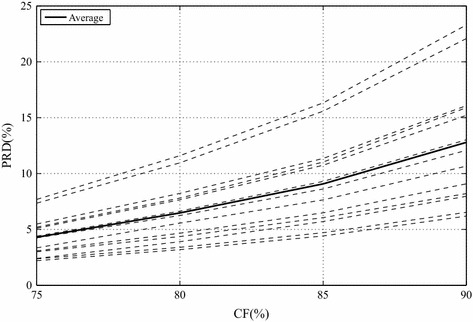
Performance of the modified two-dimensional MMP encoder with dynamic signals

**Fig. 15:**
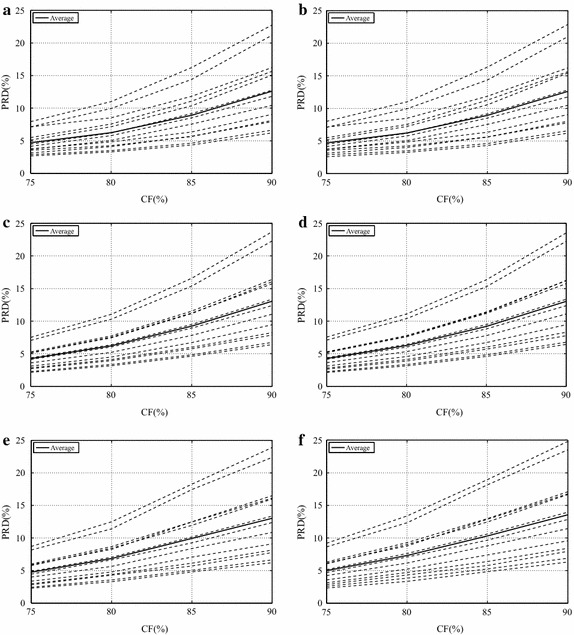
Performance of the proposed scheme regarding dynamic signals, for,** a** PDS and HEVC,** b** SbS and HEVC,** c** PDS and H.264/AVC,** d** SbS and H.264/AVC,** e** PDS and JPEG2000, and** f** SbS and JPEG2000

Results regarding the dynamic database (13 dynamic SEMG signals), concerning the proposed image encoders and preprocessing techniques, are shown in Figs. 14 and 15, along with average values in Table 3.

**Table 3:** Average PRD (%) results for the proposed methodology, regarding dynamic signals

	Compression factor			
	75 %	80 %	85 %	90 %
JPEG2000	4.997	7.326	10.357	13.769
JPEG2000 + PDS	4.743	6.834	9.927	13.025
JPEG2000 + SbS	4.996	7.312	10.314	13.552
H.264/AVC	4.510	6.564	9.630	13.486
H.264/AVC + PDS	4.256	6.214	9.236	13.056
H.264/AVC + SbS	4.270	6.262	9.194	13.043
HEVC	4.784	6.410	9.220	12.871
HEVC + PDS	4.707	6.248	8.909	12.603
HEVC + SbS	4.709	6.233	8.929	12.605
Mod. 2-D MMP	4.296	6.474	9.069	12.788

Firstly, PRD figures lower than 7 % are only achieved for CFs lower than 75 %, as shown in Figs. 14 and 15. Besides, there are no large differences between the proposed preprocessing techniques, except for some records compressed with JPEG2000.

The following discussion refers to Table 3. At a first glance, it is clear that the modified two-dimensional MMP is not as successful as in the isometric scenario. MMP overcame H.264/AVC for high CFs (85 and 90 %) and HEVC for a CF of 75 %, even when they used preprocessing techniques, and the base H.264/AVC, for the entire test CF range. Regarding the base HEVC algorithm, MMP is competitive and even provides better performance for CFs of 75, 85, and 90 %, but it is outperformed when preprocessing techniques are employed, at CFs of 80, 85 and 90 %.

With respect to JPEG2000, H.264/AVC, and HEVC, H.264/AVC + PDS provided the best results for CFs of 75 and 80 %, while HEVC + PDS overcame all other options for CFs of 85 and 90 %, as can be seen in Table 3. It seems that the relatively high-peaks existent in dynamic signals are better handled by H.264/AVC, since there are fewer prediction directions, which require fewer bits to be encoded, and PRD results tend to be reduced (since it is a relative measure), due to high sample values (peaks). As already happened, during the previous simulations, the performance of HEVC, in relation to H.264/AVC, tends to improve with higher CFs. The performance difference between the proposed preprocessing techniques was much smaller, when compared with what was obtained in the isometric scenario. In summary, the proposed schemes provided good results, even when JPEG2000 is combined with SbS, which had not happened during simulations with isometric signals.

JPEG2000 is not as competitive as in the isometric scenario and, in the present case, the combination with SbS presented a slight performance increase (see Fig. 15; Table 3). As in the previous scenario, the best results were obtained with the PDS technique. It is worth noticing that JPEG2000 + PDS outperformed H.264/AVC, even with preprocessing techniques, for a CF of 90 %, as shown in Table 3.

H.264/AVC presented the its best performance with PDS, for CFs of 75, 85, and 90 %, whereas, for a CF of 80 %, the combination with SbS produced the best result. Besides, its performance degrades for high CFs (see Fig. 15), in comparison with the other schemes.

HEVC provided competitive results, mainly for CFs greater than 80 %; however, at low CFs, it was overcome by H.264/AVC. This is an interesting result, given what was obtained with isometric signals.

As a general comment, preprocessed dynamic SEMG matrices are not as homogeneous as isometric ones, which is closely related to the original signals. Indeed, in dynamic contractions, there may be variations in speed, strength, and joint angle, which modify the signal behavior during tests. As a consequence, even the SbS technique is not able to create a high-correlated arrangement, as in the isometric case, which has an impact on the flexible prediction scheme of HEVC. In addition, this same behavior compromises the convergence of the adaptive dictionary module in MMP.

Differently from the previous simulations, no combination with preprocessing technique presented decrease in performance, when compared with the respective base algorithm. It indicates that representations created with the proposed preprocessing techniques present high correlation and compensate the additional side information. Finally, both SbS and PDS have the potential to favor the adopted compressors.

Table 4 presents a brief comparison among the proposed compression methodology and some schemes available in the literature. The ones proposed by Norris et al. [22], Berger et al. [23], and Chaffim et al. [26] employ the same algorithms used for isometric signals; however, Trabuco et al. [24] used a dynamic bit allocation scheme, based on decreasing square-root spectral shapes, for encoding transform coefficients.

**Table 4:** Average PRD (%) results for dynamic SEMG signal compression, with different schemes

	Compression factor			
	75 %	80 %	85 %	90 %
Norris et al. [22]	7.93	9.06	10.02	19.98
Berger et al. [23]	2.70	4.41	7.52	20.10
Chaffim et al. (JPEG2000) [26]	–	4.39	5.77	9.39
Chaffim et al. (H.264/AVC) [26]	4.13	5.16	7.12	11.30
Trabuco et al. [24]	4.70	5.41	6.40	8.22
JPEG2000 + PDS	4.743	6.834	9.927	13.025
H.264/AVC + PDS	4.256	6.214	9.236	13.056
H.264/AVC + SbS	4.270	6.262	9.194	13.043
HEVC + PDS	4.707	6.248	8.909	12.603
HEVC + SbS	4.709	6.233	8.929	12.605
Mod. 2-D MMP	4.296	6.474	9.069	12.788

It is important to notice that the dynamic signals used by the other schemes were obtained with different acquisition protocols. Berger et al. [23] and Trabuco et al. [24] used records acquired from the vastus lateralis of individuals riding a cycling simulator, as also done by Chaffim et al. [26], which included signals from the vastus medialis, and Norris et al. [22] employed signals representing cyclic contractions of biceps, while the signals used here were obtained from the vastus lateralis muscle, when individuals were using an isokinetic dynamometer. During the test phase, some records acquired with a cycling-based protocol were retrieved and compared with the ones used here and, after a brief analysis, the main conclusion was that the latter present greater challenges to the proposed compressors.

Keeping in mind what was discussed in the last paragraph, the proposed schemes outperformed the ones presented by Norris et al. [22] and Berger et al. [23], for entire test range and only for a CF of 90 %, respectively. Regarding the methods presented by Chaffim et al. [26], the proposed ones are competitive, at low CFs. For a CF of 75 %, the best result was provided by the method presented by Berger et al. [23], while the JPEG2000 version introduced by Chaffim et al. [26] overcame all others for CFs of 80 and 85 % and Trabuco et al. [24] provided the best result for a CF of 90 %.

**Fig. 16:**
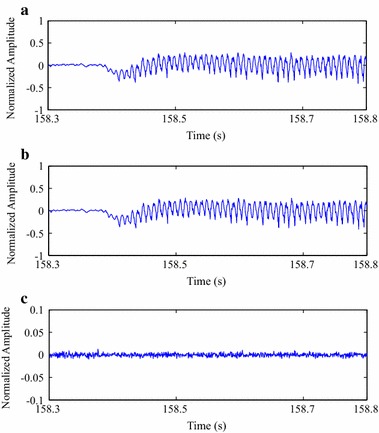
Compression result for a segment from one of the test dynamic SEMG signals, at a CF of 87.3 %:** a** original,** b** reconstructed after being encoded with the modified two-dimensional MMP, and** c** error signal

**Fig. 17:**
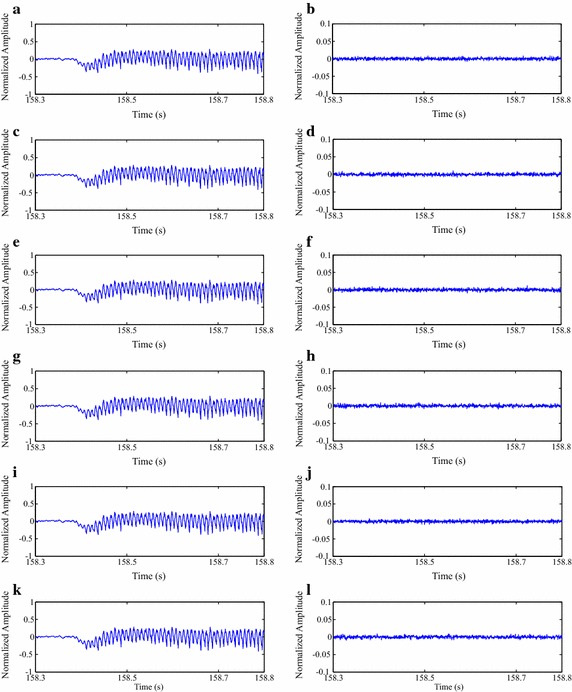
Compression result for a segment from one of the test dynamic SEMG signals, at a CF of 87.3 %:** a** signal reconstruction with SbS + HEVC and** b** error signal,** c** signal reconstruction with PDS + HEVC and** d** error signal,** e** signal reconstruction with SbS + H.264/AVC and** f** error signal,** g** signal reconstruction with PDS + H.264/AVC and** h** error signal,** i** signal reconstruction with SbS + JPEG2000 and** j** error signal, and** k** signal reconstruction with PDS + JPEG2000 and** l** error signal

In order to illustrate the quality of reconstructed dynamic SEMG signals, after compression processes with the proposed schemes, as already done for isometric records, reconstructed signal segments and the associated error are shown in Figs. 16 and 17, as well as the original portion, in Fig. 16. The proposed methodology was employed with a CF of 87.3 %, as already done for the isometric scenario. Once again, differences between original and reconstructed signals are small and the same conclusions presented for the isometric scenario apply here.

## Conclusions

This paper presents three main contributions: a two-dimensional approach based on the MMP algorithm, a new preprocessing technique called SbS, and SEMG compression results with HEVC. The MMP algorithm was adapted to work with SEMG signals and its prediction framework was replaced by an approach similar to what is used in HEVC. The proposed architecture employed three off-the-shell image compressors: JPEG2000, H.264/AVC, and HEVC, with and without the SbS and PDS preprocessing techniques.

In experiments carried out with real isometric SEMG signals, with the goal of evaluating the proposed methodology, the SbS technique was able to improve the exploitation of the intra- and intersegment dependencies of HEVC and H.264/AVC (intraframe mode), while PDS improved the performance of all chosen encoders. Furthermore, the proposed modified two-dimensional MMP algorithm presented good results and outperformed state-of-the-art methods present in the literature, for low compression factors, whereas HEVC combined with SbS achieved good results for high compression factors. Finally, JPEG2000 combined with PDS can still be regarded as an interesting option, for low compression factors.

Regarding experiments carried out with real dynamic SEMG signals, the proposed methodology do not seem to be as successful as in the isometric scenario, at least at a first glance. However, the acquisition protocol used here is different from the ones employed in the other schemes and results in signals that seem harder to compress. Nonetheless, no combination between preprocessing technique and two-dimensional encoder resulted in performance decrease. In addition, one may argue that tests with the same signals used in the other approaches may provide good results, given what was obtained with the similar schemes presented by Chaffim et al. [26]

The proposed combinations among preprocessing methods and two-dimensional encoders were efficient in encoding SEMG signals as images. The base structure can be easily implemented on different computer architectures, given that image compressors can be easily ported or may be already available. Indeed, medical applications using embedded systems can benefit from what was presented, given the potential for reducing power consumption, conserving bandwidth, and saving storage space, in applications using databases.

As future work, new preprocessing techniques for SEMG signals will be developed, which will mostly rely on spectral features, and the proposed frameworks will be adapted to other biological signals, such as ECG and EEG, which will also drive the development of other preprocessing steps. Besides, electromyographic signals acquired with electrode-arrays and high-density EMG will be investigated.
